# Regulatory mechanisms involved in muscle and bone remodeling during refeeding in gilthead sea bream

**DOI:** 10.1038/s41598-019-57013-6

**Published:** 2020-01-13

**Authors:** F. Lavajoo, M. Perelló-Amorós, E. J. Vélez, A. Sánchez-Moya, S. Balbuena-Pecino, N. Riera-Heredia, J. Fernández-Borràs, J. Blasco, I. Navarro, E. Capilla, J. Gutiérrez

**Affiliations:** 1 Department of Cell Biology, Physiology and Immunology, Faculty of Biology, University of Barcelona, Barcelona, Spain; 2 Present Address: Department of Marine Biology, Faculty of Marine Science and Technology, University of Hormozgan, Bandar Abbas, I.R. Iran; 3 Present Address: Department of Veterinary Biomedical Sciences, Western College of Veterinary Medicine, University of Saskatchewan, Saskatoon, Saskatchewan S7N 5B4 Canada

**Keywords:** Molecular biology, Physiology

## Abstract

The tolerance of fish to fasting offers a model to study the regulatory mechanisms and changes produced when feeding is restored. Gilthead sea bream juveniles were exposed to a 21-days fasting period followed by 2 h to 7-days refeeding. Fasting provoked a decrease in body weight, somatic indexes, and muscle gene expression of members of the Gh/Igf system, signaling molecules (*akt*, *tor* and downstream effectors), proliferation marker *pcna*, myogenic regulatory factors, myostatin, and proteolytic molecules such as cathepsins or calpains, while most ubiquitin-proteasome system members increased or remained stable. In bone, downregulated expression of Gh/Igf members and osteogenic factors was observed, whereas expression of the osteoclastic marker *ctsk* was increased. Refeeding recovered the expression of Gh/Igf system, myogenic and osteogenic factors in a sequence similar to that of development. Akt and Tor phosphorylation raised at 2 and 5 h post-refeeding, much faster than its gene expression increased, which occurred at day 7. The expression in bone and muscle of the inhibitor myostatin (*mstn2*) showed an inverse profile suggesting an inter-organ coordination that needs to be further explored in fish. Overall, this study provides new information on the molecules involved in the musculoskeletal system remodeling during the early stages of refeeding in fish.

## Introduction

Gilthead sea bream (*Sparus aurata* Linnaeus 1758) is one of the most important marine fish species in Mediterranean aquaculture, which has expanded over the past two decades^1^ in parallel with the scientific research and the knowledge of its physiology. Unlike mammals, fish are able to adapt to relatively long periods of starvation and it is possible to use fasting as a practice to improve product quality by reducing body lipid content, and refeeding as a way to induce compensatory growth^2–4^. Under normal feeding conditions, fish grow and store energy reserves, while in fasting body stores are mobilized to maintain life processes^5^. During fasting, the metabolism switches to a catabolic status, resulting in low growth rate, and the following refeeding reverts the situation towards a hyper-anabolic phase when organisms attempt to accelerate the growth rate^6^. Both approaches, fasting and refeeding, can be very informative in fish basic and applied research.

The effects of fasting and refeeding on body growth, metabolism, protein biosynthesis and hormonal responses have been largely studied in fish^6,7^. The muscle mass of fish species is an important tissue that considerably depends during fasting and refeeding on protein degradation and synthesis. During these stages of nutritional changes, metabolism and growth are adapted to resist the restrictions and rapidly adjust to the arrival of new nutrients. All these determine changes on the endocrine status and in the regulation of substrates mobilization by muscle and bone^6,8^. In compensatory growth studies, it has been described that refeeding stimulates proliferation of fish myogenic cells^9,10^.

The growth hormone and insulin-like growth factors (Gh/Igfs) are both, in vertebrates including fish, key factors regulating growth. Muscle and bone are widely regulated by this system and the presence of Gh and Igf1 receptors (Ghrs and Igf1Rs) and Igf isoforms as well as binding proteins (Igfbps) are well described in fish, especially in these tissues^11,12^. Moreover, in gilthead sea bream, the function of Gh/Igfs and its response to diverse conditions has been characterized and the ratio of the circulating levels of Gh and Igf1 is considered a good marker of growth quality in terms of its relation with body indexes or muscle fibers composition^12,13^.

Muscle growth is also controlled by myogenic regulatory factors (MRFs) (MyoD, Myf5, Myog and Mrf4), and the negative endogenous regulator myostatin, as well as other factors that control sequentially the process of development and growth^6,14,15^. The effects of fasting and refeeding on myogenesis have been studied in diverse fish species^6,10,16^ demonstrating the function of the different MRFs during the metabolic arrest caused with food limitation and the subsequent activation when feeding is restored. Furthermore, during fasting and refeeding, proteolytic molecules play a remarkable role to adapt to the changes in amino acids supply. This is more important in fish that have a specific and enhanced protein metabolism. The main endogenous proteolytic systems, each one performing specific degradative or regulatory functions according to the nutritional conditions are, calpains, cathepsins and ubiquitin-proteasome, all of which are well-known in fish, especially salmonids^17–19^, but also in gilthead sea bream^20–22^.

In addition to muscle, bone is also an important tissue for skeletal and locomotion functions, but also, as a reservoir of minerals that is clearly affected when nutrition is restricted. Essential during development, synchronicity between bone and muscle is required for proper musculoskeletal growth^23,24^. Besides to being induced by the Gh/Igf system, the process of osteogenesis is also regulated by skeleton-derived factors that control specific stages of osteoblasts development and bone building. Although less known in fish than in mammals, most of these molecules have been identified in gilthead sea bream^25,26^. Thus, while the Runt-related transcription factor 2 (*runx2*) and the structural molecule fibronectin 1a (*fib1a*) have a key role in the early development of the tissue, the non-collagenic molecules such as osteopontin (*op*) and osteocalcin (*ocn*), play a main role in bone maturation and matrix mineralization. However, little information exists concerning the effects of fasting and refeeding in the regulation of these molecular factors and bone development in fish species.

The objective of this study is to analyze the effects that fasting and refeeding provoke in muscle and bone in *S*. *aurata*, characterizing the expression pattern of various genes of interest of the musculoskeletal system to improve our knowledge of the regulatory mechanisms involved in these processes, and to explore their contribution to vertebrate compensatory growth. Specifically, we were interested in the early refeeding (within 24 h) when the mechanisms of tissue remodeling are triggered by the arrival of the first nutrients after 21 days of fasting, which offers an interesting scenario of regulatory molecules that restart their activity gradually after a period of latency.

## Materials and Methods

### Animals, experimental design and ethics statement

Gilthead sea bream (*Sparus aurata* L.) juveniles (initial body weight 50 ± 3 g; standard length 15.3 ± 0.68 cm; sexually immature) were obtained from a commercial hatchery (Piscimar, Burriana, Spain) and kept in the facilities of the Faculty of Biology (Universitat de Barcelona, Spain) during four weeks before acclimation period. Forty-two fish were randomly distributed into six two-hundred-liters tanks (7 fish per tank) with a recirculation system. Fish were kept at 23 ± 1 °C and a photoperiod of 12 h of light and 12 h of dark during the whole experiment. During the acclimation period (2 weeks), the fish were fed *ad libitum* twice a day with a commercial diet (Optibream, Skretting, Burgos, Spain).

After the acclimation period, a 28-day experiment was designed as previously described^27^. Briefly, it consisted in two parts: a 21-days fasting period and a 7-days refeeding period. Samples were taken at the beginning of fasting (-21 days), and at the end of it at 0, 2, 5, and 24 h and 7 days after refeeding. During the refeeding period, the fish were given the same commercial diet (Optibream, Skretting) once a day until apparent satiety. The -21 days, 24 h and 7 days samplings were performed 24 h after the last feeding. During the experiment, all the environmental conditions (temperature, photoperiod, salinity: 21 °C; 12 L: 12D; 38‰, respectively) were maintained stable.

In each sampling, six individuals (one per tank) were anesthetized and sacrificed per condition. The fish were anesthetized with MS-222 (0.08 g/L) (Sigma-Aldrich, Tres Cantos, Spain), body length (standard) and body weight were measured and blood was drawn from the caudal vein. The plasma levels of Gh and Igf1 were measured by corresponding radioimmunoassays as previously described^27^. Then, the fish were sacrificed by an overdose of the same anesthesia (0.3 g/L) and, bone from the vertebral column and skeletal white muscle from the dorsal area were dissected and snap frozen in liquid nitrogen immediately. All samples were stored at −80 °C until further analysis. This study was carried out in accordance with the recommendations of the EU, Spanish and Catalan Government-established norms and procedures. The specific protocol was approved by the Ethics and Animal Care Committee of the University of Barcelona (permit numbers CEEA 110/17 and DAAM 9488).

### Gene expression analyses

As previously described^27^, total RNA was extracted from 100 mg of skeletal white muscle and vertebral column samples using TRI Reagent Solution (Applied Biosystems, Alcobendas, Spain) and a Precellys® Evolution and Cryolys system (Bertin Technologies, Montigny-le-Bretonneux, France) for tissue homogenization. Quantity, quality and integrity were determined with a NanoDrop2000 (Thermo Scientific, Alcobendas, Spain), and a 1% agarose gel (w/v), respectively. Then, one µg RNA was treated with DNase I (Life Technologies, Alcobendas, Spain) and retrotranscribed with the Transcriptor First Strand cDNA Synthesis Kit (Roche, Sant Cugat del Vallès, Spain).

Next, the mRNA transcript levels were examined by quantitative real time PCR (qPCR) according to the requirements of the MIQUE guidelines^28^ in a CFX384^TM^ Real-Time System (Bio-Rad, El Prat de Llobregat, Spain). All reactions were performed in the conditions previously described^27^. The primers used for each tissue are listed in Supplementary Table 1. Moreover, in both tissues the reference genes elongation factor 1 alpha (*ef1α*), ribosomal protein S18 (*rps18*), beta actin (*β-actin*) and ribosomal protein L27 (*rpl27*) were analyzed and the combination of the two most stable (*ef1α* and *rps18*) was used to calculate the relative expression of the genes of interest following the Pfaffl method^29^. Both, reference genes stability and relative expression calculation were determined with the implemented Bio-Rad CFX Manager Software (v2.1).

### Protein expression analyses

Protein was extracted from 100 mg of either muscle or bone samples. Tissue homogenates were made in 1 mL of RIPA buffer supplemented with phosphatase (PMSF and NA_3_VO_4_) and protease inhibitors (P8340, Santa Cruz) using the Precellys® Evolution coupled to a Cryolys cooling system (Bertin Technologies, Montigny-le-Bretonneux, France).

Protein quantification was performed following the Bradford’s method using BSA (Sigma Aldrich, Tres Cantos, Spain) for the standard curve. Next, 15 µg of the soluble protein fraction were prepared in a loading buffer (containing SDS and β-mercaptoethanol), heated at 95 °C for 5 min and run in a 12% polyacrylamide gel. Following, the proteins were transferred overnight to Immobilon® PVDF-FL 0.2 μm Transfer Membranes (Merck Millipore Ltd., Tullagreen, Cork, Ireland), previously activated in methanol. Total transferred protein was determined by 5 min incubation with REVERT^TM^ Total Protein Stain (LI-COR, Lincoln, Nebraska, USA) and reading at 700 nm using the Odyssey Fc Imaging System (LI-COR). Membranes were blocked in Odissey Blocking Buffer (diluted 1:1 in TBS) (LI-COR) for 1 h at room temperature, and then overnight at 4 °C and in agitation in the corresponding diluted primary antibody. The primary antibodies used were as follows: Goat polyclonal anti cathepsin L (anti-Ctsl D-20 antibody; catalog no. sc-6501), goat polyclonal anti-cathepsin D (anti-Ctsd; catalog no. sc-6486) all from Santa Cruz Biotechnolgy (Santa Cruz, California, USA), rabbit polyclonal anti-phospho Akt (cat- no. 9271), anti-total Akt (cat. no. 9272) and anti-phospho Tor (cat. no. 2971) from Cell Signaling Technology (Beverly, MA, USA) and anti-total Tor (cat. no. T2949) from Sigma-Aldrich (Tres Cantos, Spain). All these antibodies have been previously demonstrated to cross-react successfully with the proteins of interest in gilthead sea bream^22,23,30^. Subsequently after washing with TBS-T, the membranes were incubated with the corresponding secondary antibodies: goat anti-rabbit (Cat. No. 925-32211, Servicios Hospitalarios) or donkey anti-goat (Cat. No. 925-32214, Servicios Hospitalarios) diluted at 1:10000 in the same blocking buffer. After incubation, membranes were washed with TBS-T and fluorescence signal was measured at 800 nm using the Odyssey Fc Imaging System (LI-COR). Stripping was performed using a commercial stripping buffer (NewBlot PVDF 5X Stripping Buffer (LI-COR). Detailed information about Western blot membranes distribution can be found in Supplementary Information 3.

### Statistical analyses

The data obtained was analyzed using IBM SPSS Statistics vs. 22 and is presented as a mean ± standard error of the mean (SEM). Normality and homogeneity of the variances were tested with the Shaphiro-Wilk’s test and the Levene’s test respectively. When the data did not show a normal distribution or homoscedasticity, were transformed by logarithm. Differences among groups were tested by one-way analysis of variance (ANOVA) followed by Tukey HSD or LSD, as *post-hoc* tests. In the case that normality and/or homoscedasticity were not found even after logarithmic data transformation, the non-parametric Kruskal-Wallis test or the Dunnett’s T3 as *post-hoc*, were used. The confidence interval for all analyses was set at 5%.

## Results

### Somatic parameters and GH/IGF-I ratio

As shown in Supplementary Table 2, fasting for 21 days provoked an arrest of growth (i.e. body weight and condition factor, CF) that tended to recover after 7 days refeeding. Similarly, hepatosomatic index (HSI) felt down significantly during fasting to reach normal values after 7 days refeeding. Moreover, the viscerosomatic index (VSI) was also significantly reduced by fasting, and refeeding induced an acute increase to recover the basal levels after 1 day. Fasting resulted also in a high Gh/Igf1 ratio that only recovered basal values after 7 days refeeding. In fact, as we previously reported^27^, plasma Gh levels raised from 0,77 ± 0,03 to 32,77 ± 0,79 ng/ml in response to 21 days of fasting with a progressive decrease to basal levels with refeeding; while circulating Igf1 levels showed an inverse pattern, decreasing from 15,35 ± 2,51 to 5,9 ± 1,32 ng/ml after fasting and recovering only after a long-term refeeding.

### Muscle responses to fasting and refeeding

#### GH and IGF family

**Figure 1:**
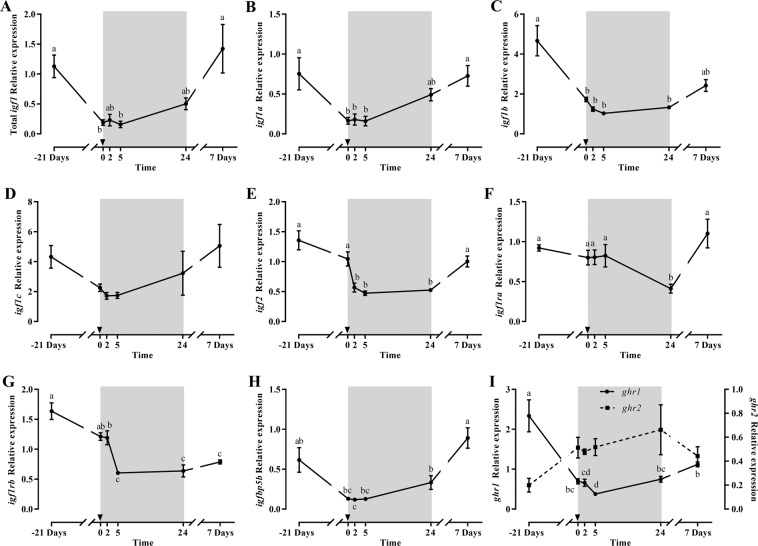
Relative gene expression of skeletal white muscle total *igf1* (**A**), *igf1a* (**B**), *igf1b* (**C**), *igf1c* (**D)**, *igf2* (**E**), *igf1ra* (**F**), *igf1rb* (**G**), *igfbp5* (**H**) and *ghr1* and *ghr2* (**I**) in gilthead sea bream during the fasting and refeeding experiment. The postprandial period is shown in grey and the time in hours. Data are shown as means ± SEM (n = 6). Letters indicates significant differences (p < 0.05) by one-way ANOVA, LSD and Tukey HSD test.

Total *igf1* gene expression showed a significant decrease after 21 days of fasting recovering basal levels by day 7 of refeeding (Fig. 1A); the same effect was observed for the *igf1* splice variants *igf1a* and *igf1b*, but not for *igf1c* (Fig. 1B–D). The *igf2* (Fig. 1E) profile was similar although a significant decrease was observed from 2 h refeeding and basal levels recovered at 7 days refeeding. Between the *igf1rs*, *igf1ra* was not affected by fasting but decreased significantly at 24 h refeeding recovering later (Fig. 1F), while *igf1rb* decreased during fasting, and continued dropping at 2 and 5 h refeeding reaching then significantly the lowest levels that remained low after 7 days refeeding (Fig. 1G). The expression of the *igfbps*, *igfbp1* and *igfbp4* was unaltered (data not shown), but *igfbp5b* decreased significantly at 2 h refeeding, and recovered basal levels 7 days after refeeding (Fig. 1H). Concerning *ghrs*, they showed an inverse profile, with *ghr1* decreasing significantly during fasting and at 2 and 5 h refeeding, to increase at 1 and 7 days refeeding; while *ghr2*, although not significantly, progressively increased through the experiment up to 24 h of refeeding, decreasing afterwards at day 7 (Fig. 1I).

#### Signaling molecules

**Figure 2:**
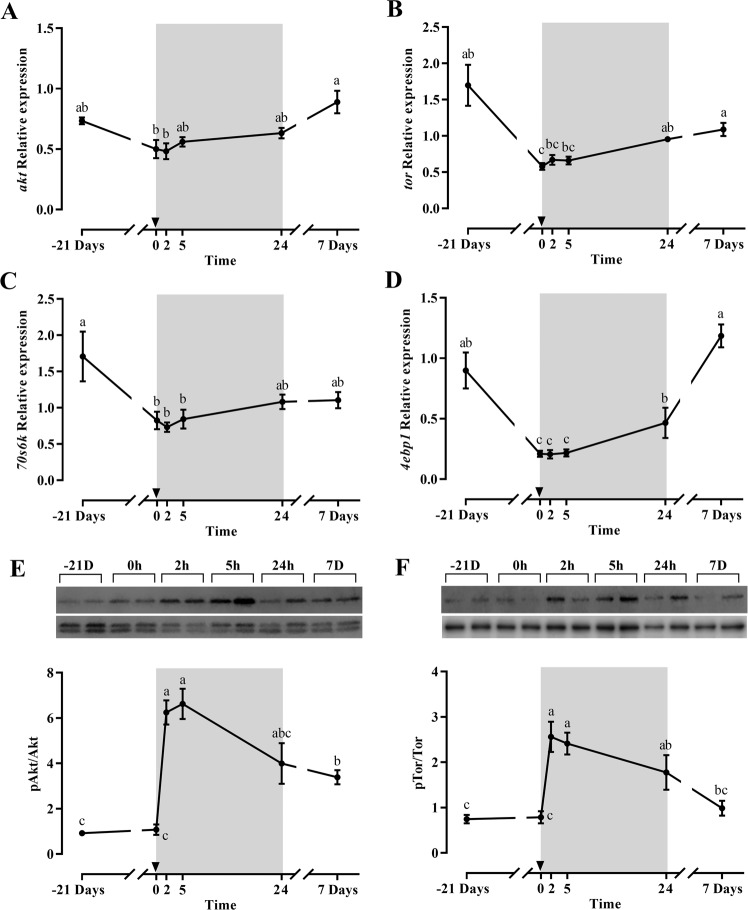
Relative gene expression of skeletal white muscle *akt* (**A**), *tor* (**B**), *70s6k* (**C**) and *4ebp1* (**D**) and representative blot and densiometric analysis of the phosphorylation ratios of Akt (**E**) and Tor (**F**) in gilthead sea bream during the fasting and refeeding experiment. For the Western blots, the same membranes cropped in two were used to analyze Tor (top part) and Akt (bottom part). The phosphorylated forms were analyzed first and after stripping, the corresponding total forms were determined in the same membranes. The intensity of the phosphorylated form was normalized by its total form, and the intensity of each specific band was normalized by the total transferred protein for the corresponding well. The postprandial period is shown in grey and the time in hours. Data are shown as means ± SEM (n = 6). Letters indicates significant differences (p < 0.05) by one-way ANOVA, LSD and Tukey HSD test.

The mRNA levels of *akt*, *tor*, and the downstream molecules *70s6k* and *4ebp1* (Fig. 2A–D) showed a similar gene expression profile. With the exception of *akt*, all molecules exhibited a significant decrease with fasting and a progressive increase during the refeeding period, significant at day 7 for *akt*, *tor* and *4ebp1*. Interestingly the postprandial response for Akt and Tor was much faster when their phosphorylation was studied (Fig. 2E,F), being significantly increased at 2 and 5 h refeeding compared to time zero and decreasing later at 1 and 7 days. *foxo3* did not show any significant difference along the experiment (data not shown).

#### Muscle growth-related factors

**Figure 3:**
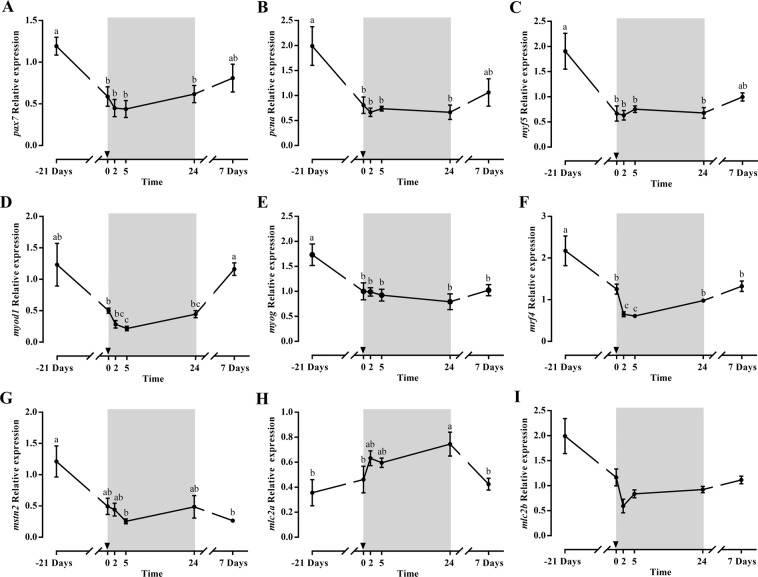
Relative gene expression of skeletal white muscle *pax7* (**A**), *pcna* (**B**), *myf5* (**C**), *myod1* (**D**), *myog* (**E**), *mrf4* (**F**), *mstn2* (**G**), *mlc2a* (**H**) and *mlc2b* (**I**) in gilthead sea bream during the fasting and refeeding experiment. The postprandial period is shown in grey and the time in hours. Data are shown as means ± SEM (n = 6). Letters indicates significant differences (p < 0.05) by one-way ANOVA, LSD and Tukey HSD test.

The profiles of *pax7* and *pcna* expression were similar and correlated very well with those of *myf5* and *myod1*, decreasing significantly during fasting and partially recovering after 7 days refeeding (Fig. 3A–D). The expression of other myogenic genes also decreased significantly during fasting or early refeeding (*myog*, *mrf4*, *mstn2*) (Fig. 3E–G), or showed similar tendencies (*myod2*, *mstn1*, data not shown). At 7 days of refeeding, *mrf4* started to increase again while *myog* and *mstn2* remained at low levels. Moreover, the structural myosin light chains, *mlc2a* and *mlc2b* showed an inverse profile with a significant increase for *mlc2a* at 24 h refeeding, returning to basal levels at day 7, and maintained expression for *mlc2b* (Fig. 3H–I).

#### Proteolytic systems’ genes

**Figure 4:**
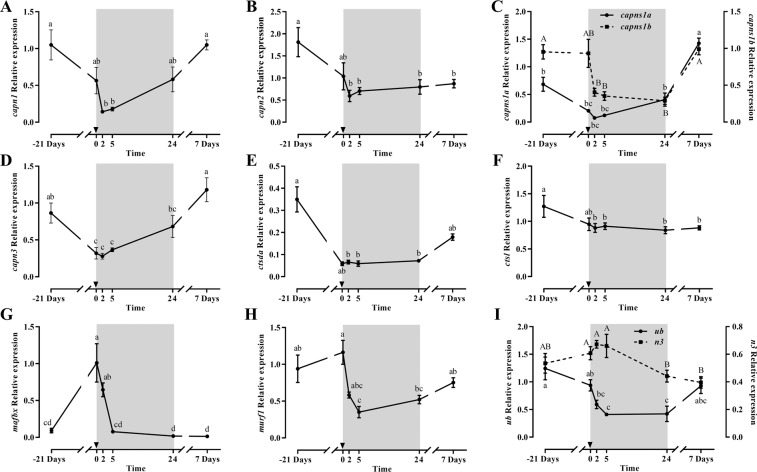
Relative gene expression of skeletal white muscle *capn1* (**A**), *capn2* (**B**), *capns1a* and *capns1b* (**C**), *capn3* (**D**), *ctsda* (**E**), *ctsl* (**F**), *mafbx* (**G**), *murf1* (**H**) and *ub* and *n3* (**I**) in gilthead sea bream during the fasting and refeeding experiment. The postprandial period is shown in grey and the time in hours. Data are shown as means ± SEM (n = 6). Letters indicates significant differences (p < 0.05) by one-way ANOVA, LSD and Tukey HSD test.

**Figure 5:**
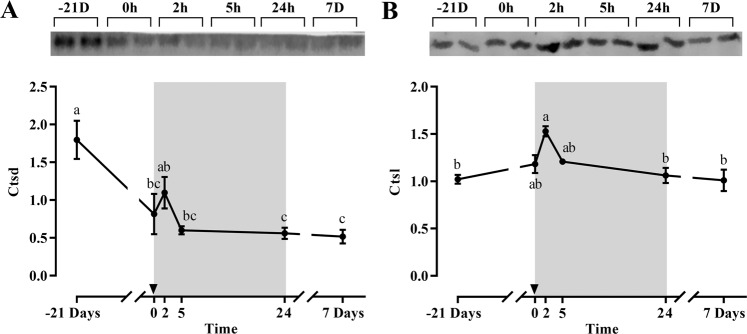
Representative blot and densiometric protein levels of skeletal white muscle Ctsd (**A**) and Ctsl (**B**) in gilthead sea bream during the fasting and refeeding experiment. Each protein was analyzed in cropped membranes of different Western blots along with other proteins (data not shown). The intensity of each specific band was normalized by the total transferred protein. The postprandial period is shown in grey and the time in hours. Data are shown as means ± SEM (n = 6). Letters indicates significant differences (p < 0.05) by one-way ANOVA, LSD and Tukey HSD test.

Most of the proteolytic genes expression was significantly downregulated during fasting or early refeeding, recovering basal levels after 7 days refeeding (Fig. 4). This was the case for *capn1*, *capns1b*, *capn3*, *ctsda* and *ub*, and a similar tendency was observed for *capns1a*. Moreover, *capn2* and *ctsl* expression was also significantly downregulated with fasting but after 7-days refeeding were not able to recover basal values. It is remarkable how *mafbx* and *murf1* increased or were maintained after 21 days of fasting to significantly decrease after 5 or 24 h refeeding, reaching basal levels at the end of the experiment, while *n3* showed only minor changes on expression along the whole experiment (Fig. 4G–I). Regarding protein expression, only Ctsd decreased significantly with fasting, but both Ctsd and Ctsl presented similar patterns postprandially with a single peak at 2 h (Fig. 5).

### Bone responses to fasting and refeeding

#### GH and IGF family

**Figure 6:**
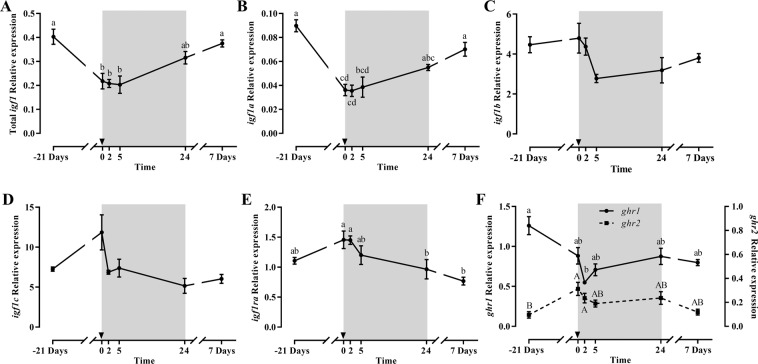
Relative gene expression of bone total *igf1* (**A**), *igf1a* (**B**), *igf1b* (**C**), *igf1c* (**D**), *igf1ra* (**E**) and *ghr1* and *ghr2* (**F**) in gilthead sea bream during the fasting and refeeding experiment. The postprandial period is shown in grey and the time in hours. Data are shown as means ± SEM (n = 6). Letters indicates significant differences (p < 0.05) by one-way ANOVA, LSD and Tukey HSD test.

Bone total *igf1* and *igf1a* mRNA levels showed significant lower values after the 21 days of fasting, returning to basal levels at 24 h and 7 days refeeding (Fig. 6A,B). Differently, *igf1b* and *igf1c* expression was not significantly affected by fasting or refeeding (Fig. 6C,D); and *igf1ra* expression was maintained at fasting but significantly decreased at 1 and 7 days refeeding (Fig. 6E). Neither i*gf1rb* nor *igfbp*s were affected by fasting or refeeding (data not shown). *ghrs* expression presented, as observed in muscle, a reverse profile. Thus, while *ghr1* expression decreased significantly during fasting and early refeeding, *ghr2* expression increased significantly during the same period. Then, both *ghrs* returned to initial levels at 24 h or 7 days refeeding (Fig. 6F).

#### Bone-related genes

**Figure 7:**
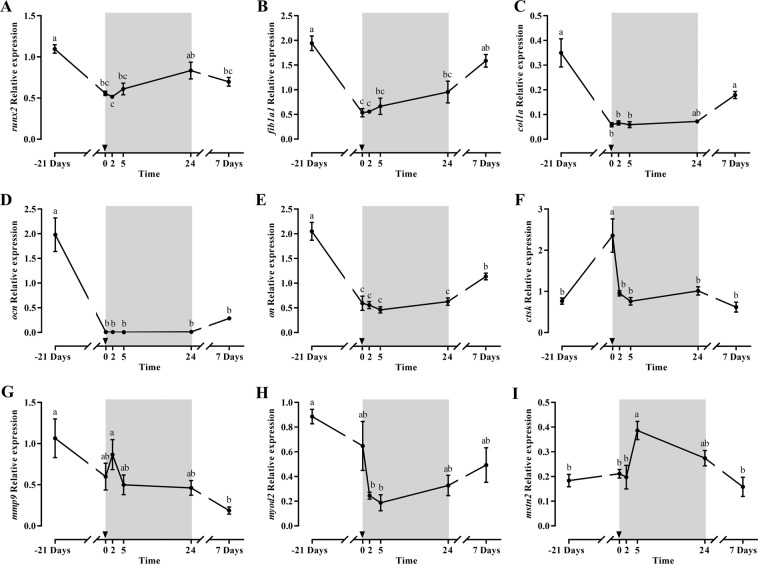
Relative gene expression of bone *runx2* (**A**), *fib1a* (**B**), *col1a1* (**C**), *ocn* (**D**), *on* (**E**), *ctsk* (**F**), *mmp9* (**G**), *myod2* (**H**) and *mstn2* (**I**) in gilthead sea bream during the fasting and refeeding experiment. The postprandial period is shown in grey and the time in hours. Data are shown as means ± SEM (n = 6). Letters indicates significant differences (p < 0.05) by one-way ANOVA, LSD and Tukey HSD test.

The expression of the osteogenic factors *runx2*, *fib1a*, *col1a1*, *ocn* and *on* showed a significant diminution during fasting or early refeeding (Fig. 7A–E). Then, *runx2*, *fib1a* and *col1a1* recovered the basal mRNA levels after 1 or 7 days of refeeding, while *ocn* and *on* presented a similar tendency but without reaching basal levels and still remaining low at day 7.

Concerning the osteoclastogenic genes, the expression level of *ctsk* increased after 21 days of fasting, to recover the basal levels at 2 h refeeding, while *mmp9* showed a tendency to decrease at 21 days fasting, peak at 2 h refeeding to then significantly decrease at 7 days refeeding (Fig. 7F,G). *trap* showed a very similar profile to *mmp9* but without significant changes throughout the experiment (data not shown).

#### Muscle growth-related factors

First, *pax7* gene expression was studied in bone samples to confirm purity of the tissue and the results obtained showed very low mRNA levels, close to undetectable showing a basal line without changes throughout the experiment (data not shown). Then, the other myogenic genes studied in bone (i.e. *myod1*, *myod2*, *mrf4*, *mstn1* and *mstn2*) did not show significant responses to either fasting or refeeding (data not shown) with the exception of *myod2* and *mstn2* (Fig. 7H,I). *myod2* expression decreased during fasting showing significant low levels at early refeeding (2 and 5 h), and recovered basal values at the end of the experiment; *mstn2* showed an almost contrary profile, since it was stable during fasting but peaked at 5 h postprandial to progressively return to basal values at 24 h and 7 days refeeding.

## Discussion

Fish are well adapted to resist fasting periods and this characteristic has been used to study the mechanisms that regulate the balance between anabolism and catabolism in these vertebrates^7^. The present study continues the previous one already published by Perelló-Amorós and coworkers^27^ in which the function of ghrelin during fasting and refeeding was characterized in gilthead sea bream. That paper reported also the effects on *gh* and *igf1* pituitary and liver expression, hormone plasma levels and the fish biometric indexes. Briefly, fasting provoked an increase in ghrelin plasma levels and a decrease on liver *igf1* expression, while refeeding reversed progressively the condition. In the present study, we focused on the Gh/Igf axis and other regulatory systems in muscle and bone, providing valuable information on the regulation of the musculoskeletal system, especially in bone, a tissue still poorly explored in fish. Refeeding reversed the fasting condition and the sequential sampling permitted to analyze how regulatory mechanisms returned gradually to the growth condition. Although the absence of a pair-fed control group does not allow differentiating among refeeding and postprandial effects, the present data suggest that early refeeding after 21-days fasting amplifies the responses likely occurring postprandially at an attenuated magnitude. Thus, the experimental model would emphasize the main steps of skeletal muscle remodeling that take place in gilthead sea bream when food is restored.

Igfs play an important role on growth regulation in vertebrates^12,31–33^ and during adverse conditions are downregulated to preserve the survival of the animal over the body growth. Gh secretion is dependent of nutrients and both, Gh and Igf1 regulate metabolism in fish^7,34^. In gilthead sea bream, 21 days of fasting resulted in a general decrease in the muscular gene expression of total *igf1* and its splice variants. This is in agreement with the significant decrease of liver total *igf1* expression previously described in the same animals^27^, indicating altogether that both, systemic and local Igf1 production, might contribute to arrest muscle growth under these catabolic conditions. Similarly, Peterson and coworkers^33^ in channel catfish (*Ictalurus punctatus*) reported that fasting for 30 days reduced fish weight by approximately 60% and decreased *igf1* mRNA in muscle, and this has been also seen in other fish species^11,35^. This decrease in Igf1 during a nutrient-starvation period can stop muscle proliferation and development as observed in an *in vitro* muscular model of gilthead sea bream under specific amino acids limitation (i.e. lysine)^36^. In the current study, both *igfrs* were affected differently; thus, *igf1ra* did not respond to fasting but to refeeding, recovering the expression levels at day 7, while the decreased expression levels of *igf1rb* in fasting and early refeeding were maintained until the end of the experiment. Such a differential response has been reported in several species using *in vivo* or *in vitro* models^8,10,32,36,37^ and suggests a functional split between both *igfrs* isoforms. Furthermore, these findings point out that during a fasting or a low food intake period, Igf1 production decreases, but contrarily, some of the *igfrs* isoforms can be maintained, suggesting a rise in Igf sensitivity^8,32,38,39^.

Muscle *igfbps* also responded differently to the treatment and while expression of *igfbp1* or *igfbp4* was unchanged, *igfbp5* significantly decreased in fasting and recovered progressively after 7 days of refeeding. This is in agreement with the anabolic function attributed to *igfbp5*, which has been identified as a good growth marker in gilthead sea bream^40^. Similar responses were detected in some species^41,42^; but not in others^35,43,44^, although such a decrease is analogous to the Igfbp3 plasma levels determined in fasted coho and chinook salmon^45–47^, suggesting a similar response of these two *igfbps* to long term food deprivation in different fish species. Thus, Igfbps in teleost are differently affected by the various metabolic conditions^42^ and in the case of gilthead sea bream muscle, *igfbp5b* seems to be the most sensitive form to food availability. Moreover, *ghrs* expression showed in this study a dual role, with the decrease of *ghr1* until 5 h refeeding and a partial increase at 24 h and 7 days refeeding and the increase of *ghr2* expression up to 24 h refeeding. These patterns coincide with the differential function attributed to each receptor in gilthead sea bream and other fish species, being Ghr1 anabolic and Ghr2 catabolic^23,48^. Thus, in gilthead sea bream the growth enhancement caused by sustained exercise was followed by an increase in *ghr1* expression and a decrease in *ghr2* expression in muscle^40^. Similarly in this species, Gh treatment induced in muscle an increase of *ghr1* expression but not *ghr2*^23^. In summary, fasting depresses the components involved in growth promotion (*ghr1*, *igf1*, *igf1rb*, *igfbp5*), while refeeding reverts progressively the situation to activate muscle recovery.

Next, the expression of key elements of the main signaling pathways regulating protein synthesis and muscle growth (*akt* and *tor*) was significantly diminished during fasting. Such a scenario has been also observed in different fish species exposed to food deprivation^11,22^. The present study also demonstrates the sequential activation of those pathway components with the recovery of nutrition; however, it is very interesting to compare such a progressive response in gene expression with the quick phosphorylation of Akt and Tor within 2 h post-feeding. Similar responses were previously observed in fine flounder (*Paralichthys adspersus*)^11^, rainbow trout^19,49^ and even in gilthead sea bream both *in vivo*^50^, as well as *in vitro*^30^. Thus, the comparison of *akt* and *tor* mRNA levels with their corresponding protein phosphorylation status during fasting and refeeding in the present study corroborates the different timely regulation of these pathways at protein and gene levels. Nevertheless, according to our results, it has to be taken into account that while refeeding is likely a major factor in the gene/protein expression and/or phosphorylation responses, they may also occur to some degree (especially those related with protein activation), during the introduction of nutrients in a common postprandial period. Therefore, future studies should consider these overlapping responses to specifically identify the dynamic nature of how nutrients regulate these processes in fish.

The information on MRFs under nutritional restriction in gilthead sea bream is scarce and current results could be useful to understand muscle growth regulation in such catabolic situations. Similarly to that observed in cultured muscle cells with lysine deficiency^36^, in this study the expression of *myod1*, *myf5*, *myog*, *mrf4* and *mstn2* decreased during fasting or early refeeding, while late refeeding recovered basal expression of *myods* and *myf5*. Thus, making noticeable that the sequence of MRFs up-regulation during refeeding follows the same characteristic order as during myogenesis activation^15^. The parallelism between the profile of *myf5*, *myod1* and *myod2* with *pcna* or *pax7* agrees with the involvement of these MRFs in the first stage of myogenesis. Interestingly, similar patterns of recovery after starvation have been reported for several MRFs, mostly *myod* and *myog*, in salmonids^16,32,51,52^ and in juvenile Nile tilapia (*Oreochromis niloticus*)^6^; overall, confirming the beginning of muscle remodeling at that early stage of refeeding. On the other hand, *mstn2* expression decreased with fasting and maintained significant low levels after 7 days refeeding, which is in agreement with previous results found in rainbow trout^52^ and sea bass (*Dicentrarchus labrax*)^53^. Thus refeeding provoked a clear down-regulation of *mstn* gene expression to favor muscle recovery, in parallel to the increase observed of *pax7*, *pcna*, and MRFs expression.

Concerning muscle structural components, *mlc2a* and *mlc2b* showed an inverse profile, with *mlc2a* expression not changing with fasting but showing a significant increase at 24 h refeeding to return to basal levels at day 7. Bower and Johnston^37^ also found in Atlantic salmon an increase of *mlc2* after 14 days of refeeding, and previous studies in gilthead sea bream^22,23,54^ showed that an increase in specifically, *mlc2a* expression, indicates a condition that favors muscle proliferation. In this sense, in the present study the *mlc2a* peak at 24 h refeeding paralleled the tendency to enhance the expression of *pax7*, *pcna*, *tor* and the MRFs involved in early myogenesis (*myod2* and *myf5*), facilitating the muscle to grow during this period of compensatory growth.

Several members of the main proteolytic systems have been characterized and their responses upon different experimental challenges investigated in different fish species^18,55–60^, including gilthead sea bream^20–22^; however, due to the different experimental conditions (i.e. duration of periods, fish age, etc.) among studies, it is difficult to reach clear explanation of their respective roles. In the present study, two different expression patterns were observed concerning proteolytic genes; those that were downregulated during fasting and recovered with refeeding, like calpains and some cathepsins, in a similar trend to that observed for *igf1* or MRFs; and those belonging to the ubiquitin-proteasome system, like *murf1* or *mafbx*, which increased in response to fasting and decreased with refeeding.

Regarding the calpains, in agreement with our findings, channel catfish fasted for 35 days presented a strong down-regulation of *capn1* and *capn3* genes, which recovered basal expression after a refeeding period^58^. Contrarily, Salmerón and coworkers^20^ did not find significant effects on calpains expression in gilthead sea bream and suggested that these molecules could have a secondary role in the adaptation to food deprivation; and in fact, observed later that *ctsd* and *ctsl* increased significantly under the same conditions^21^, similarly as observed in halibut for *ctsb* and *ctsd*^8^. Although we did not observe an increase in the gene expression of *ctsda* or *ctsl* in this study, amino acids limitation in *in vitro* myocytes upregulated both cathepsins expression^61^. Moreover, in the present study, Ctsl protein expression increased in fasting or early refeeding while Ctsd decreased, suggesting this different response certain distribution of their regulatory functions. Concerning the ubiquitin-proteasome system, in a previous experiment in gilthead sea bream, 30 days of fasting upregulated the expression of the same genes that were increased in the present study^21^. Different authors have found similar responses of these genes with fasting^19,44,50,60,62^ and, specific amino acids limitation increased also the expression of *mafbx* and *murf1*^61^. Interestingly, results were also supported at a protein level *in vivo*, since fasted rainbow trout muscle presented an increase in the total amount of polyubiquitinated proteins^19^.

As an overview, in the present fasting and refeeding model, it seems that there is a clear difference between the expression of genes of the three major proteolytic pathways, being the ubiquitin-proteasome system the one that may have a stronger role in response to food restriction. Refeeding reverts the condition with calpains recovering high basal levels, while cathepsins present a variable role depending on the molecule considered. All this suggests a coordinated distribution of the proteolytic functions during the mobilization of reserves (fasting) and the remodeling associated with the compensatory muscle-growth induced by refeeding.

The skeletal system has multiple physiological functions in vertebrates^63^, and the high incidence of skeletal deformities is still an important bottleneck for the sustainability of aquaculture^64^. Information about the effects of fasting and refeeding on bone has been mainly studied in cellular-boned fish, like the rainbow trout^65,66^, while in fish species with acellular bone such as gilthead sea bream knowledge is limited. In mammals, it is well known that Igfs are important mediators of bone growth^67,68^, being present at high concentrations in the bone matrix and, any deficiency on the Igf genes affecting skeletal growth^69^. Igfs stimulate differentiation of osteoblasts regulating the balance between bone accretion and resorption, which occurs throughout life^70^. So, in a fasting condition, or under severe chronic undernutrition, Igf1 concentrations decrease in association with low bone turnover and significant bone loss^71,72^. In fish, early studies also demonstrated the growth stimulatory effect of Igf1 in branchial arches of Japanese eel (*Anguilla japonica*)^69^ and more recently, in an *in vitro* model of bone-derived cells from vertebra of gilthead sea bream, Igf1 as well as insulin, were demonstrated to stimulate cell proliferation^70^.

In the current study, the effects of fasting and refeeding in bone were, in terms of the Gh/Igf system, similar to those found in muscle. Thus, the expression of *ghr1* and *ghr2* was inverse at fasting and refeeding, but the profiles were similar between the two tissues, supporting the conservation of Ghrs’ role as well as their coordination in muscle and bone to adapt to the changes in alimentary conditions. About peptides, fasting resulted in a decrease of total *igf1* and *igf1a* expression, supporting reduced bone growth during catabolic conditions, while the other splice variants or the *igfbps* did not respond to the treatments. In agreement to these data, several authors previously demonstrated the proliferative effects of Igf1 *in vitro* in gilthead sea bream osteoblasts, and in embryonic zebrafish ZF-4 cells^73–75^. Thus, the bone total *igf1* downregulated expression observed in fasting is parallel to the decrease in *igf1* and *pcna* expression in muscle and, the general arrest of growth. On the other hand, during refeeding the upregulated expression of total *igf1* and *ghr1* in bone as in muscle, pointed out also to the importance of the coordination between the two tissues in this period, for the harmonic growth of the muscle-skeletal system for the fish to properly increase its size.

Several genes regulate the osteogenic process, and *runx2* is the key transcription factor determining bone lineage and inducing the expression of genes more involved in matrix production and mineralization (i.e. *fib1a*, *col1a1*, *ocn* and *on*)^26,76^. The results of the current study showed that during the fasting period, all osteogenic genes decreased in agreement with results on biometric indexes, MRFs in muscle and, the Gh/Igf system. During starvation, active osteoblasts were not observed in rainbow trout pharyngeal bone, and in Nile tilapia, an imbalance between bone formation and resorption (i.e. osteoblasts *versus* osteoclasts activity) was reported, resulting in reduced bone mass^65,77^. Moreover, Van der Velde and coworkers^78^ suggested also that during fasting, the orexigenic hormone ghrelin increases osteoclastogenesis in mice. In the animals of the present study, ghrelin levels increased during fasting to diminish in an acute way at 5 h post-feeding^27^. Thus, during the fasting period, the levels of circulating ghrelin can contribute to arrest the osteogenic process increasing bone degradation. The decrease in osteoclastic activity fits well with the significant enhancement of *ctsk* expression observed after 21 days of fasting and its down-regulation in refeeding, since Ctsk is secreted by osteoclasts and is an important factor of bone resorption^79^. In this sense, the present study demonstrates such an equal role in gilthead sea bream.

In osteoblast-like cell lines of this species, fish serum is more suitable than bovine serum to induce bone formation causing changes in *col1a*, *ocn* and *on* gene expression^80^. In the present study, the expression of osteogenic genes was restored with refeeding in a sequential order, being *runx2* the first gene upregulated while the other genes followed progressively (*fib1a*, *col1a1*, *on* and *ocn*) in a sense that resembled the osteogenic process^26^, indicating that bone growth and matrix mineralization were being reinstated. In agreement, in goldfish (*Carassius auratus*), it was found that while *col1a* continually increased after day 7 of scale regeneration, *ocn* increased only after day 14^81^. Based on our results, 7 days of refeeding were not enough to recover the latest genes *on* and *ocn*, like in muscle for *myog* or *mrf4*, suggesting that both tissues recovered in parallel to guarantee an harmonic growth; besides indicating that more than 7 days of refeeding are necessary for complete restoration of the musculoskeletal system anabolism.

The expression of different myogenic genes in mammalian bone is known and their role in coordinating both skeletal tissues has been described^82,83^ but these studies in fish are scarce. In the present study, *pax7*, a marker of muscle satellite cells^15,84^, was evaluated in order to verify the purity of the tissue, which was corroborated since *pax7* expression was undetectable in the bone samples. Then, the gene expression of other MRFs was determined. García de la serrana and coworkers^85^ in a transcriptional study in gilthead sea bream, found for the majority of genes analyzed a faster and more pronounced response to leucine injection in skeletal muscle than in bone. A similar tendency was found in the present study when comparing both tissues, since *myod2* expression showed in bone still low levels at 5 and 24 h or 7 days refeeding, while in muscle the levels were already recovered by the end of the period studied. On the other hand, bone *mstn2* expression, showed an opposite response compared to muscle with a significant increase at 5 h post-feeding, which could be inhibiting bone growth at this specific stage. In humans, the role of myostatin in bone resorption and rheumatoid arthritis pathology was recently demonstrated^82^; thus, the inhibitory function of Mstn2 in fish, could serve also in this early refeeding period as a regulator to an overly rapid recovery of bone growth. In fact, the inter-tissue inhibitory role of myostatin has been already described in mammals^83,86^ serving as a coordinator among skeletal tissues. This function still needs to be demonstrated in fish, but myostatin could contribute not only to control muscle mass, but also to coordinate the growth of both tissues avoiding miss-matches and potentially reducing the appearance of skeletal deformities.

**Figure 8:**
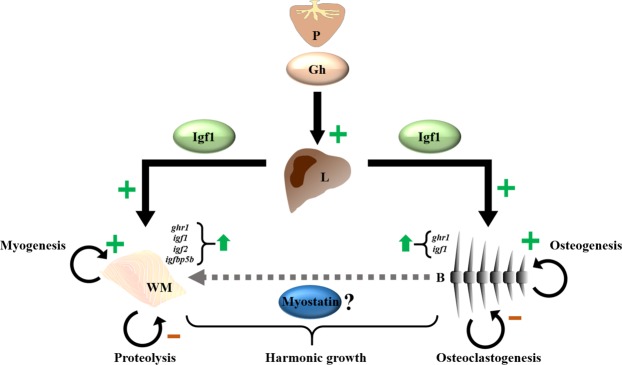
Schematic representation of the proposed changes occurring during early refeeding in gilthead sea bream. The Gh/Igf system recovers the synthetizing role with the Gh plasma levels still elevated activating now the hepatic expression/secretion of *igf1*, in parallel with the progressive up-regulation of the anabolic system components (*ghr1*, *igf1*, *igf2* and *igfbp5b*). This condition contributes to the activation of the myogenic (*pax7*, *myf5*, *myod1* and *mrf4*) and osteogenic (*runx2*, *fib1a*, *col1a1* and *on*) genes, while downregulates the proteolytic (*mafbx* and *murf1*) and osteoclastogenic (*ctsk*) genes in muscle and bone, respectively. This early stage of refeeding may require a fine regulation of the different molecules involved, being myostatin a good candidate for bone and muscle crosstalk to assure harmonic musculoskeletal growth. P: pituitary; L: liver; WM: white muscle; B: bone.

In summary, 21 days of fasting in gilthead sea bream depressed Igf1 as well as most of the synthesis, myogenic and osteogenic mechanisms at the same time that activated several proteolytic molecules from the ubiquitin-proteasome system to mobilize muscle resources. Refeeding triggered rapidly compensatory mechanisms starting with the Igf1 system, and progressively activated the regulatory factors of myogenesis and osteogenesis in a sequence that repeats the processes of development and growth in either tissue, overall restoring proper musculoskeletal growth (Fig. 8). Furthermore, the expression in bone of myostatin suggests an interesting tissue coordinative function that deserves future investigation in this and other fish species. All this can help to better understand the role of these regulatory factors and their crosstalk in the early phase of somatic growth recovery after a period of food limitation.

## Supplementary information

Supplementary Information. 

## Data Availability

All data generated or analysed during this study are included in this published article (and its Supplementary Information files).
