# Supplementary material for: Regulatory mechanisms involved in muscle and bone remodeling during refeeding in gilthead sea bream
Source: Sci Rep. 2020 Jan 13;10:184. doi: 10.1038/s41598-019-57013-6 (PMC6957526; doi:10.1038/s41598-019-57013-6)
Supplement: Supplementary file 1 — Supplementary Information. [file 41598_2019_57013_MOESM1_ESM.pdf]

Regulatory mechanisms involved in muscle and bone remodeling during refeeding in gilthead sea bream

Lavajoo, F.[§1], Perelló-Amorós, M.[§], Vélez, E.J.[2], Sánchez-Moya, A., Balbuena-Pecino, S., Riera-Heredia, N., Fernández-Borràs, J., Blasco, J., Navarro, I., Capilla, E.[¥] and Gutiérrez J.[¥*]

Department of Cell Biology, Physiology and Immunology, Faculty of Biology, University of Barcelona, Barcelona, Spain

[§] Equal contribution; [¥] Equal contribution

[1]Present address: Department of Marine Biology, Faculty of Marine Science and Technology, University of Hormozgan, I. R. Iran

[2]Present address: Department of Veterinary Biomedical Sciences, Western College of Veterinary Medicine, University of Saskatchewan, Saskatoon, Saskatchewan S7N 5B4, Canada

*Corresponding author: Joaquim Gutiérrez

Department of Cell Biology, Physiology and Immunology, Faculty of Biology, University of Barcelona, Av. Diagonal 643, 08028, Barcelona, Spain. Tel: (+34) 934021532, E-mail: jgutierrez@ub.edu

Supplementary Table 1: Sequences, annealing temperatures (Ta) and GenBank accession numbers of the primers used for *Sparus aurata* real-time quantitative PCR.

	Gene	Sequences	Ta (ºC)	Accession Number
GH/IGF family	*igf1a*	F: AGGACAGCACAGCAGCCAGACAAGAC<br>R: TTCGGACCATTGTTAGCCTCCTCTCTG	60	AY996779
	igf1ab	F: AGTCATTCATCCTTCAAGGAAGTGCATCC<br>R: TTCGGACCATTGTTAGCCTCCTCTCTG	60	EF688015
	igf1abc	F: ACAGAATGTAGGGACGGAGCGAATGGAC<br>R: TTCGGACCATTGTTAGCCTCCTCTCTG	60	EF688016
	igf2	F: TGGGATCGTAGAGGAGTGTTGT<br>R: CTGTAGAGAGGTGGCCGACA	60	AY996778
	igf1ra	F: AGCATCAAAGACGAACTGG<br>R: CTCCTCGCTGTAGAAGAAGC	60	KT156846
	igf1rb	F: GCTAATGCGAATGTGTTGG<br>R: CGTCCTTTATGCTGCTGATG	55	KT156847
	igfbp1	F: AGTGCGAGTCCTCTCTGGAT<br>R: TCTCTTTAAGGGCACTCGGC	60	KM522771
	igfbp4	F: TCCACAAACCAGAGAAGCAA<br>R: GGGTATGGGGATTGTGAAGA	60	F5T95CD02JMZ9K
	igfbp5b	F: TTTCTCTCTCGGTGTGC<br>R: TCAAGTATCGGCTCCAG	60	AM963285
	ghr1	F: ACCTGTCAGCCACCACATGA<br>R: TCGTGCAGATCTGGGTCGTA	60	AF438176
	ghr2	F: GAGTGAACCCGGCCTGACAG<br>R: GCGGTGGTATCTGATTCATGGT	60	AY573601
Signaling	*akt*	F: GCTCACCCCACTCTTCAGAC<br>R: AAATTGGGAAATGTGCTTGC	60	AY996779
	tor	F: CAGACTGACGAGGATGCTGA<br>R: AGTTGAGCAGCGGGTCATAG	60	EF688015
	70s6k	F: GCACCAGAAAGGCATCATCT<br>R: AAGGTGTGGGTCACTGTTCC	60	EF688016
	4ebp1	F: CCAACCTGCGACTCATCTCT<br>R: GTTCCTCTCATCCTCCCACA	60	KM522771
	foxo3	F: CAGCAGCCTGGAGTGTGATA<br>R: CCAGCTCTGAGAGGTCTGCT	60	
Muscle growth-related	*pax7*	F: ATGAACACTGTCGGCAACG<br>R: AGGCTGTCCACACTCTTGATG	64	JN034418
	pcna	F: TGTTTGAGGCACGTCTGGTT<br>R: TGGCTAGGTTTCTGTCGC	60	AY550963.1
	myf5	F: CTACGAGAGCAGGTGGAGAACT<br>R: TGTCTTATCGCCCAAAGTGTC	64	JN034420
	myod1	F: TTTGAGGACCTGGACCC<br>R: CTTCTGCGTGGTGATGGA	60	AF478568.1
	myod2	F: CACTACAGCGGGGATTCAGAC<br>R: CGTTTGCTTCTCCTGGACTC	60	AF478569
	myog	F: CAGAGGCTGCCCAAGGTGGAG<br>R: CAGGTGCTGCCCGAACTGGGCTCG	68	EF462191
	mrf4	F: CATCCCACAGCTTTAAAGGCA<br>R: GAGGACGCCGAAGATTCACT	60	JN034421
	mstn1	F: GTACGACGTGCTGGGAGACG<br>R: CGTACGATTCGATTCGCTTG	60	AF258448.1
	mstn2	F: ACCTGGTGAACAAAGCCAAC<br>R: TGCGGTTGAAGTAGAGCATG	60	AY046314
	mlc2a	F: GCCCCATCAACTTCACCGTCTTT<br>R: GGTTGGTCATCTCCTCAGCGG	60	AF150904

	mlc2b	F: TCCCTTTGCTATTCTGCCTTC<br>R: AAATCAGCCCTATTCCCCATA	60	FG618631
Proteolytic systems	*capn1*	F: CCTACGAGATGAGGATGGCT<br>R: AGTTGTCAAAGTCGGCGGT	56	KF444899
	capn2	F: ACCCACGCTCAGACGGCAAA<br>R: CGTTCCCGCTGTCATCCATCA	61	KF444900
	capn3	F: AGAGGGTTTCAGCCTTGAGA<br>R: CGCTTTGATCTTTCTCCACA	56	ERP000874
	capns1a	F: CGCAGATACAGCGATGAAAA<br>R: GTTTTGAAGGAACGGCACAT	56	KF444901
	capns1b	F: ATGGACAGCGACAGCACA<br>R: AGAGGTATTTGAACTCGTGGAAG	56	ERP000874
	ctsda	F: CCTCCATTCACTGCTCCTTC<br>R: ACCGGATGGAAAACTCTGTG	56	AF036319
	ctsl	F: ACTCCTTGGGCAAACACA<br>R: CCTTGAACTTCCTCTCCGT	54	DQ875329
	ub	F: ACTGGCAAGACCATTACCTT<br>R: TGGATGTTGTAGTCGGAAAG	54	KJ524459
	murf1	F: GTGACGGCGAGGATGTGC<br>R: CTTCGGCTCCTTGGTGTCTT	60	FM145056
	mafbx	F: GGTCACCTGGAGTGGAAGAA<br>R: GGTGCAACTTTCTGGGTTGT	60	ERA047531
	n3	F: AGACACACACTGAACCCGA<br>R: TTCCTGAAGCGAACCAGA	54	KJ524458
Bone-related	*runx2*	F: ACCCGTCCTACCTGAGTCC<br>R: AGAAGAACCTGGCAATCGTC	60	JX232063
	fib1a	F: CGGTAATAACTACAGAATCGGTGAG<br>R: CGCATTTGAACTCGCCCTTG	60	FG262933
	col1a1	F: GAGATGGCGGTGATGTGGCGGAGTC<br>R: GCCTGGTTTGGCTGGATGAAGAGGG	68	DQ324363
	ocn	F: TCCGCAGTGGTGAGACAGAAG<br>R: CGGTCCGTAGTAGGCCGTGTAG	56	AF048703
	on	F: AGGAGGAGGTCATCGTGGAAGAGCC<br>R: GTGGTGGTTCAGGCAGGGATTCTCA	68	AY239014
	ctsk	F: AGCGAGCAGAACCTGGTGGAC<br>R: GCAGAGTTGTAGTTGGGGTCGTAG	60	DQ875329
	mmp9	F: ATTCAGAAGGTGGAGGGAGCG<br>R: CATTGGGGACACCACCGAAGA	60	AM905938
Reference	*ef1a*	F: CTTCAACGCTCAGGTCATCAT<br>R: GCACAGCGAAACGACCAAGGGGA	60	AF184170
	rps18	F: GGGTGTTGGCAGACGTTAC<br>R: CTTCTGCCTGTTGAGGAACCA	60	AM490061.1
	b-actin	F: TCCTGCGGAATCCATGAGA<br>R: GACGTCGCACTTCATGATGCT	60	X89920
	rpl27	F: AAGAGGAACACAACTCACTGCCCCAC<br>R: CTTCTGCCTGTTGAGGAACCA	68	AY188520

Supplementary Table 2: Body weight (BW), body length (BL), condition factor (CF), hepatosomatic index (HSI), viscerosomatic index (VSI) and ratio of Gh and Igf1 plasma levels along the fasting and refeeding experiment. Data are shown as means ± SEM (n=6). Letters indicate significant differences ($p < 0.05$) by one-way ANOVA, LSD and Tukey HSD test.

	**-21 Days**	**0h**	**2h**	**5h**	**24h**	**7 Days**
**BW (g)**	53,9 ± 3,0	47,2 ± 3,2	49,4 ± 1,9	50,8 ± 2,3	46,8 ± 1,7	54,4 ± 3,5
**BL (cm)**	12,9 ± 0,27	13,1 ± 0,34	13,1 ± 0,21	13,2 ± 0,24	13,0 ± 0,18	13,4 ± 0,29
**CF**	2,49 ± 0,05 (A)	2,11 ± 0,06 (B)	2,22 ± 0,05 (B)	2,23 ± 0,03 (B)	2,14 ± 0,04 (B)	2,27 ± 0,03 (B)
**HSI**	1,27 ± 0,11 (B)	0,58 ± 0,06 (C)	0,58 ± 0,06 (C)	0,68 ± 0,06 (C)	0,81 ± 0,08 (C)	1,66 ± 0,1 (A)
**VSI**	0,66 ± 0,13 (AB)	0,36 ± 0,11 (C)	0,64 ± 0,13 (A)	0,37 ± 0,13 (AB)	0,3 ± 0,09 (AB)	0,55 ± 0,06 (AB)
**[Gh]/[Igf1]**	0,06 ± 0,08 (B)	2,97 ± 0,80 (A)	3,4 ± 0,23 (A)	2,85 ±1,13 (A)	3,24 ± 1,04 (A)	0,89 ± 0,48 (AB)

Supplementary information 3 - Western blot full length gels: the six samples of each time group were evenly distributed in three gels resulting in 2 samples/gel/group. Due to that in each western blot, different proteins were analyzed, it was necessary to crop the membranes in order to incubate them with different primary antibodies. Tor (289 kDa MW) and Akt (60 kDa MW) phosphorylated and total forms, were analyzed in the same western blot by cutting the membranes in two. The top part was used to blot Tor antibodies and the bottom part for Akt. Phosphorylated forms were analyzed first and after stripping, the corresponding total forms were determined in the same membranes. Moreover, Ctsd (33 kDa MW) and Ctsl (23 kDa MW) were analyzed in cropped membranes of different western blots each along with other proteins that were unsuccessful (data not shown).

Western blot membranes 1, 2 and 3 top: pTor

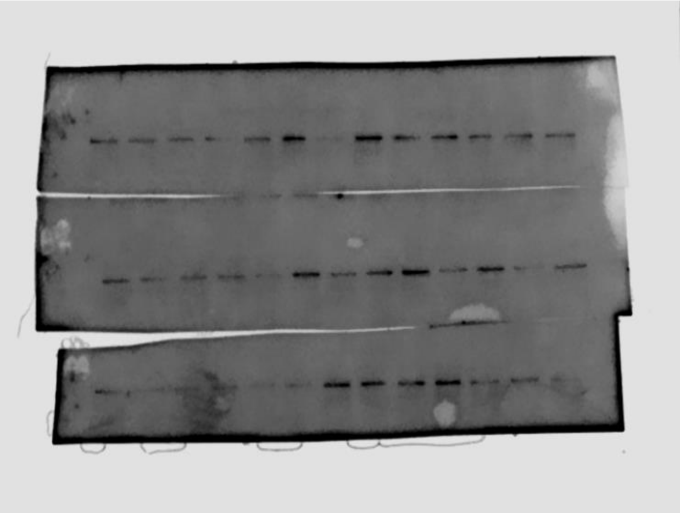

Western blot membranes 1, 2 and 3 top: Total Tor

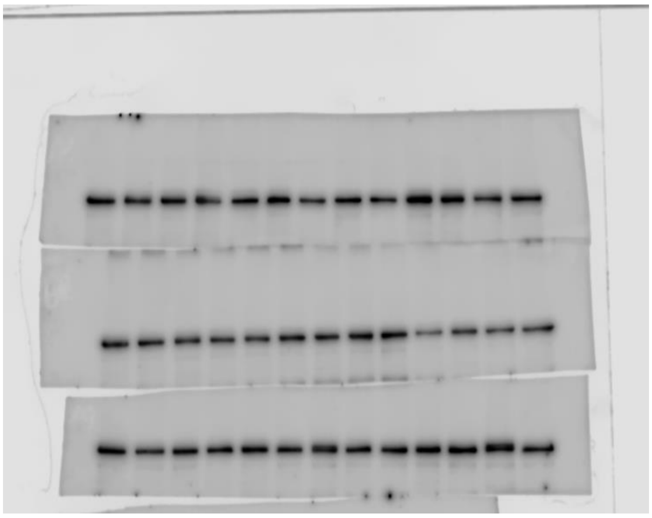

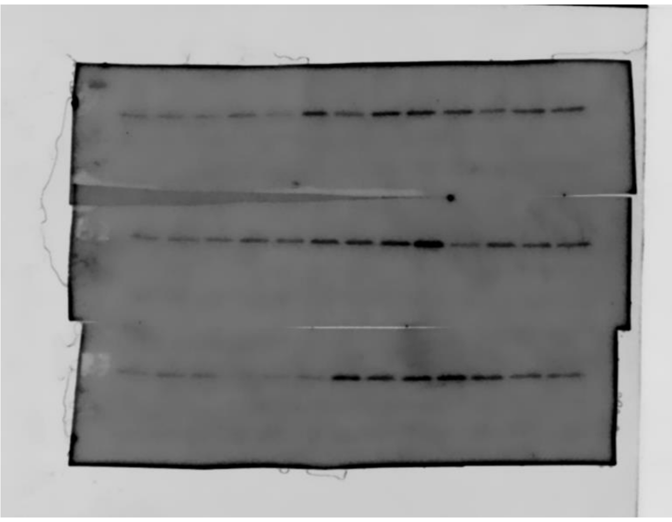

Western blot membranes 1, 2 and 3 bottom: pAkt

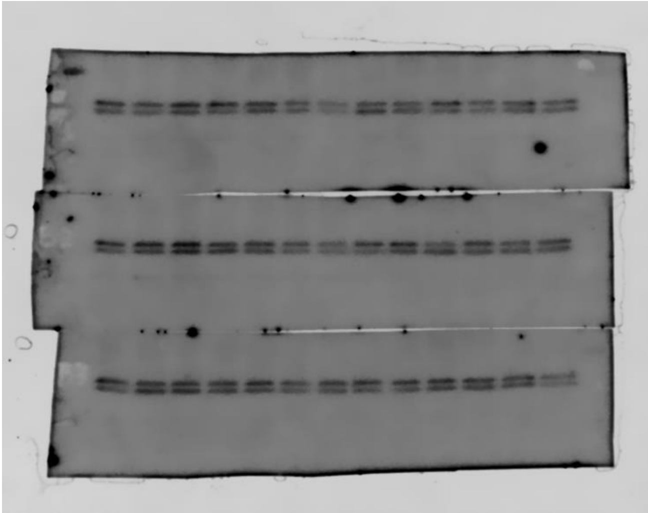

Western blot membranes 1, 2 and 3 bottom: Total Akt

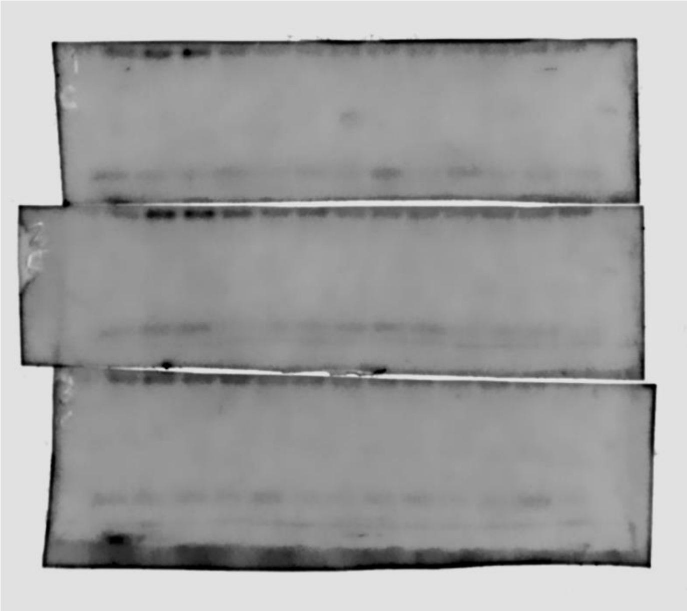

Western blot membranes 4, 5 and 6: Ctsd

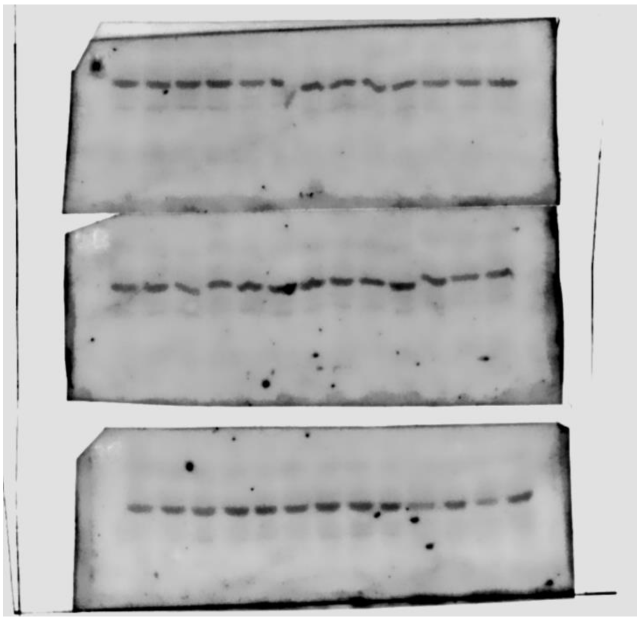

Western blot membranes 7, 8 and 9: Ctsl
